# Densification: Hyaluronan Aggregation in Different Human Organs

**DOI:** 10.3390/bioengineering9040159

**Published:** 2022-04-05

**Authors:** Antonio Stecco, Mary Cowman, Nina Pirri, Preeti Raghavan, Carmelo Pirri

**Affiliations:** 1 Rusk Rehabilitation, New York University School of Medicine, New York, NY 10016, USA; antonio.stecco@nyulangone.org; 2 Department of Biomedical Engineering, New York University Tandon School of Engineering, New York, NY 10016, USA; mary.cowman@nyu.edu; 3 Department of Medicine—DIMED, School of Radiology, Radiology Institute, University of Padua, 35122 Padova, Italy; nina_92_@hotmail.it; 4 Department of Physical Medicine and Rehabilitation and Neurology, Johns Hopkins University School of Medicine, Baltimore, MD 21205, USA; praghav3@jhmi.edu; 5 Department of Neurosciences, Institute of Human Anatomy, University of Padova, 35121 Padova, Italy

**Keywords:** densification, hyaluronan, aggregation, liver, kidney, fascia, blood vessels, eye, skin, lung, muscle

## Abstract

Hyaluronan (HA) has complex biological roles that have catalyzed clinical interest in several fields of medicine. In this narrative review, we provide an overview of HA aggregation, also called densification, in human organs. The literature suggests that HA aggregation can occur in the liver, eye, lung, kidney, blood vessel, muscle, fascia, skin, pancreatic cancer and malignant melanoma. In all these organs, aggregation of HA leads to an increase in extracellular matrix viscosity, causing stiffness and organ dysfunction. Fibrosis, in some of these organs, may also occur as a direct consequence of densification in the long term. Specific imaging evaluation, such dynamic ultrasonography, elasto-sonography, elasto-MRI and T1ρ MRI can permit early diagnosis to enable the clinician to organize the treatment plan and avoid further progression of the pathology and dysfunction.

## 1. Introduction

The importance of hyaluronan (HA) at the cellular level is well known. HA can be produced by many cell types (fibroblasts, stellate cells, cancer associated fibroblasts, fasciacytes) [1], although mesenchymal cells are believed to be the predominant source of HA. Indeed, a burst of HA synthesis occurs just prior to mitosis, enabling some cells to become dissociated from neighboring cells and to lose the adhesion from their surrounding extracellular matrix (ECM) in preparation for division [2]. However, its role at the level of the organ and organism level has been less studied [3]. HA originally thought to be a mere “space-filler” in the ECM is now known to have important roles both structurally and as a signaling molecule [4]. HA fragments are able to stimulate several cell surface receptors, such as CD44, RHAMM, LYVE-1, HARE, layilin and Toll-4, acting as signaling pathways for gene expression regulation [5]. Furthermore, other receptors as TSHR [6], molecules as leukoregulin [7] and emmprin [8], and conditions as hypoxia [9], can stimulate the synthesis of hyaluronan [10,11]. HA fragments can promote tumor cell growth through CD44-dependent tyrosine kinase signal in tumor cells [5,12,13,14]. HA has complex biological roles that have catalyzed clinical interest in several fields of medicine, such as ophthalmic surgery, articular pathologies, cutaneous repair, skin remodeling, vascular prosthesis, adipose tissue engineering, nerve reconstruction and cancer therapy [15,16]. HA is present in many strains of bacteria and in all vertebrates, in which it is localized in the embryonic tissues and soft connective tissue ECM [15].

HA is the simplest of the glycosaminoglycans (GAGs) composed of a linear non-sulfated polymer of up to 20,000 repeats of its disaccharide unit D-glucuronic acid and N-acetyl-D-glucosamine [17]. Due to the carboxyl groups in the molecule, HA is negatively charged and highly hydrophilic, being able to hold water molecules up to 1000-fold of its molecular weight [17]. HA is produced by HA synthases (HAS1–3) in the plasma membrane of different cells (fibroblasts, stellate cells, cancer associated fibroblasts, fasciacytes), for then be secreted out of the cell membrane [17]. HAS1 and HAS2 are both able to produce HA of 2–4 × 106 Da, but HAS2 is the main HA synthase in the skin [17]; while HAS3 produces HA of 0.4–2.5 × 105 Da [4]. The turnover of HA occurs rapidly and its half-life varies from 12–24 h in the skin to a few minutes in the bloodstream [18]. The different HA molecular sizes can display different and sometimes opposing biological actions. In fact, the equilibrium between HA synthesis and degradation has a key role in determining the molecular weight (MW) of HA and, consequently, its properties [19,20]. Therefore, HA has a key role not only physiologically, such as in ECM hydration and regulation of tissue homeostasis and resistance to compressive forces, but also in pathological conditions [15]. At high molecular weights, HA is able to form a viscous network, and by interacting with many proteoglycans, such as aggrecan, gives rise to molecular composites that occupy a huge volume and are responsible for the gel state of the matrix. These large complexes of HA and HA-binding proteoglycans crosslink with other matrix proteins, such as collagen, and result in the formation of super-molecular structures which increase tissue stiffness [21]. Excessive accumulation of HA in a spread surface can dramatically increase its viscosity and alter its lubricating properties [22]. At high concentrations, HA behaves similar to a non-Newtonian fluid and becomes more viscous [23], because the HA chains entangle, contributing to the hydrodynamic properties of the solution. Hyper-viscous ECM increases the passive resistance to movement, and can reduce force transmission during movement, with the consequent stiffness [24]. Stecco et al. identified this phenomenon in fascial tissue defining it as “densification”: a high viscous state of ECM due to HA super-aggregation with a decreased water binding capacity [25]. The purpose of this narrative review is to outline the mechanisms underlying increase of the tissue stiffness with HA accumulation and aggregation, identify which organs are affected and the clinical consequences in each of them.

## 2. Materials and Methods

**Figure 1:**
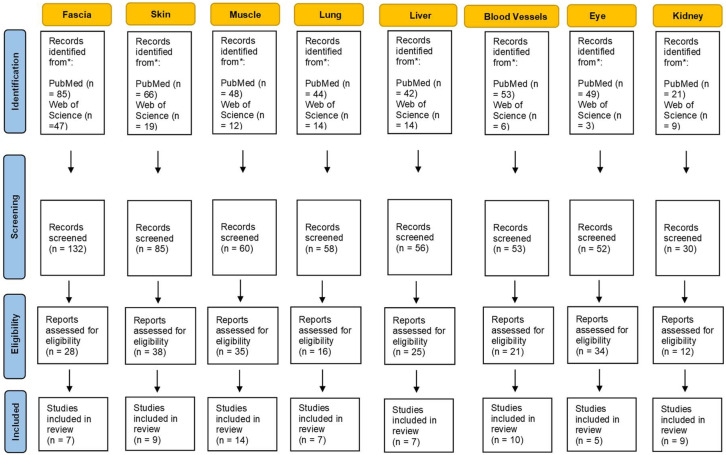
Flowchart of selection process and identification of eligible studies.

A substantial literature search was conducted to review the literature from 1960 through October 2021. Two electronic databases, i.e., PubMed and Web of Science were used to identify all relevant English publications without any category restriction. The MeSH keywords used were: “hyaluronan aggregation”, “hyaluronic acid aggregation”, “hyaluronan densification” and “stiffness”. The search was extended through the reference lists of the recruited texts. Relevant secondary references were also captured. The authors looked for papers discussing hyaluronan aggregation and stiffness in different extra-articular anatomical structures. Different organs and tissues were found including liver, kidney, eyes, skin, blood vessels, lung, muscle and fascia. Two reviewers (CP and NP) independently selected the articles by reading the titles and abstracts. A third reviewer (AS) finalized the selection in case of disagreement. After the selection, each title/abstract/full text was independently evaluated by each of the authors. The following data were recorded; any discrepancies were resolved by agreement among the authors. The flowchart of the study is shown in Figure 1.

## 3. Results

### 3.1. Fascia

Superficial, deep, and visceral fasciae are composed of both dense (collagen fibers type I and III) and loose connective tissue layers (adipose cells, HA and other GAGs).

HA, a major constituent of fascia, is responsible for its specific physical properties such as thixotropy and viscoelasticity [26]. The viscosity coefficient of HA is not constant or linearly viscous—its viscosity is reduced during loading (shear thinning), whereas at rest, the HA returns to a more viscous state [27]. Chytil et al. [28] demonstrated that chains of high molecular weight HA (10^6^–10^7^ Da), at lower shear stress levels, are efficient in re-associating in their previous superstructure, after the load has been removed. Aggregated HA increases the viscosity of the loose connective tissue, within the fasciae layers, compromising the mechanical behavior of deep fascia [29].

Stecco et al. [30] showed ECM accumulation within the fascial layers in subjects suffering from myofascial pain syndrome localized in the neck area. After the application of 45 min of manual therapy (once a week for 3 weeks) the amount of ECM within the fascial layers decreased with a correlated increase in cervical spine range of motion as well as decrease in pain sensation during cervical movements. Menon RG et al. [31] showed T1ρ imaging evidence of an alteration in the antebrachial fascia in individuals with lateral epicondylitis or tennis elbow. The fascia appeared to demonstrate higher concentrations of unbound water allowing an indirect quantification of GAG content and its physical state. After the application of 3 sessions of manual therapy (each of them of 40 min) the quantity and quality of the GAGs decreased. The authors speculate that increased T1ρ values prior to manual therapy reflect increased deposits of aggregated and poorly hydrated GAG and HA with poorer lubricant properties. This peculiar state of isolated self-aggregated GAG or HA without water may explain the stiffness [32] within the deep fascia that limits proper gliding between its layers or with the underlying muscle.

### 3.2. Skin

In the skin is present more than 50% of the entire body HA [33]. In fact, in the human dermis ECM, HA is one of the most abundant molecules (~0.5 mg/g wet weight), while in the epidermis is ~0.1 mg/g [33]. It is also known that in the dermis and in the epidermis the HA turnover rate are, respectively, a half-life of <1 day and 2–4 h [17]. The combination of hyaluronidase (HYAL) 1 and 2 mediates this rapid clearance [17]. 

Since HA exits in both free [34] and tissue receptor-bound [35] forms, HA crosslinking with matrix proteins determines the formation of supermolecular structures increasing tissue stiffness [4]. Older skin shows a higher association of HA with other tissue proteins [36], resulting in reduced free HA [36]. The same authors reported that HA concentration is 4.3 and 6.7 times higher, respectively, in adults and senescent material, compared to fetal skin. This suggest that, with increasing age, HA became progressively more tightly bound [36]. Even in photoaged skin, as maybe result of abnormal accumulation, an increase in dermal HA was observed that [21]. 

### 3.3. Muscle

HA acts as a lubricant within the endomysium, perimysium, and epimysium to aid the sliding of muscle fibers during movement [37], and its layer thickness is continuously remodeled in response to mechanical stimuli. Histological studies using animal models have shown that joint immobilization leads to the accumulation of hyaluronan in the skeletal muscle [38]. Following a stroke, catabolic pathways are activated in muscles that enhance muscle wasting and atrophy leading to ECM volume increase relative to muscle fibers [39]. An imbalance ECM turnover, as ensues of muscle paralysis and immobility, can result in increasing HA production and/or reduced degradation. Accumulation of HA in the ECM can increase its fluid viscosity causing lack of gliding between muscle fibers, reduced force transmission and consequently increased resistance or stiffness during attempted movement [40]. 

This phenomenon can also explain why immobility reduces range of motion, as noted in the ankles and feet when taking the first few steps out of bed in the morning [41]. Herda et al. [42] reported that, following two minutes of dynamic stretching, passive resistive torque and passive stiffness decreased. This indicated modifications in the viscoelastic properties of the muscular-tendon unit. Similarly, Nordez et al. [43] have reported that, during cyclic stretching protocols, viscosity plays a major role in passive stiffness changes. Other authors explained how an increase in temperature, not only reduce the muscles viscous resistance [44], but also the passive resistive torque and the muscular-tendon unit stiffness [45]. A recent case series proved the efficacy of intramuscular hyaluronidase injections for reducing muscle stiffness, and increasing passive and active range of motion in the post-stroke arm, with over three months lasting effects [40]. The same authors have demonstrated the presence of high concentrations of HA in muscles affected by central neurological injury using T1ρ MRI mapping which is sensitive to the chemical exchange of large macromolecules such as hyaluronan with protons in bulk water [46]. Prolonged immobility also initiates a complex pathologic pathway resulting in fibrogenesis [47]. In fact, an alteration in the structure and function of the muscle, defined as contracture, can be due to an excess of ECM HA that was then replaced by collagen, leading to permanent and irreversible thickening of the endomysium and perimysium [48]. 

### 3.4. Lung

Nettelbladt et al. [49] found that normal lung tissue shows positive staining for HA in the basal membrane and in the submucosal tissue of bronchi and bronchioles and the adventitia of arteries and veins. In contrast, in the areas treated with bleomycin, the alveoli demonstrated increased accumulation of HA with positive HA staining of the alveolar interstitial tissue in injured areas with markedly thickened alveolar septa. HA in the normal rat lung is also bordered to peribronchial and perivascular spaces, while it is not visible in the alveolar tissue [49]. Hence it was suggested that the accumulation of HA has a role in alveolar interstitial edema. In the early phase of bleomycin-induced lung injury, there is a considerable but transient accumulation of HA in the alveolar space that provides a matrix for later deposition of denser structures such as collagen. These results were confirmed by Juul et al. [50] who saw, as response to fibrogenic stimulus in lung exposed to bleomycin injury, an increased deposition of HA. In previous studies of the process of healing in damaged tissue, it has been documented that an early accumulation of HA precedes scar formation [51]. 

Bronchoalveolar lavage studies in humans have demonstrated highly increased lavage fluid HA concentrations in interstitial diseases, extrinsic alveolitis [52], idiopathic lung fibrosis, and adult respiratory distress syndrome [53]. In addition, Collum SD et al. [54] revealed that hyaluronan fragments resulted in increased human pulmonary artery smooth muscle cell stiffness and proliferation, demonstrating that elevated hyaluronan is a pathological process that modulated pulmonary hypertension associated with lung fibrosis that can be treated by inhibiting hyaluronan synthesis.

### 3.5. Liver

HA is a major component of the extracellular matrix of the liver [55]. In the fibrotic liver the quantity of ECM can be up to eight-fold higher than that of the normal liver [56]. Fibrosis involves histological and molecular re-arrangement of various types of collagens, proteoglycans, glycoproteins and HA [57]. For this reason, HA level is one of the best predictors of liver fibrosis and is directly linked to modifications in ECM turnover during fibrogenesis [58]. It was proven a continuous hepatic stellate cell (HSC) activation, and therefore increased HA synthesis during chronic liver inflammation [59]. As consequence of liver cell injury, together with HA level serum rising, a transformation of stellate cells into myofibroblasts occur, releasing various ECM components as elastin, collagens, glycoproteins, and proteoglycans [59]. The same Authors proved that HSC activation is the result of an im balance between ECM synthesis and degradation [59]. Sakakibara et al. reported hyaluronic acid and dermatan sulfate accumulation in the liver parenchymal cells after 10 days incubation [60]. Similar results in vivo were found by Koizumi T et al. [61] who show how chronic hepatic damage gave rise to an increase of HA and dermatan sulfate with time as fibrogenesis advanced.

The liver is also the primary organ responsible for HA degradation [59]. Both the increased synthesis and decreased clearance during fibrogenesis increases HA concentration [62], making HA a candidate for measuring liver fibrosis.

According to Nobili et al. [63], serum HA > 1200 ng/mL makes the absence of fibrosis unlikely (7%, 95% CI: 1% to 14%), while serum HA > 2100 ng/mL makes significant fibrosis very likely (89%, 95% CI: 75% to 100%). In several others studies, HA turned out to be the best class I biomarker of fibrosis having an area under curve (AUC) of 0.97, a sensitivity of 86–100%, and specificity of about 88% in investigations of cirrhosis due to non-alcoholic fatty liver disease [64] and other etiologies [65]. In addition, Kaneda et al. [66] have selected HA as a predictive marker for severe fibrosis, defining 42 ng/mL of HA serum the 100% predictive cut-off value. This value is also associated with an optimal combination of sensitivity (100%, 95% confidence interval [CI] 90–100%) and specificity (89%, 95% CI: 80–94%).

### 3.6. Blood Vessels

HA is one of the most prevalent GAGs in the vascular extracellular matrix. Abnormal HA accumulation within blood vessel walls is prominent in most vascular pathological conditions such as atherosclerosis and restenosis [67,68]. HA is up-regulated in areas of vascular injury and increases the migration and proliferation of vascular smooth muscle cells (VSMCs), which are key events in the progression of atherosclerosis and vascular narrowing [69]. VSMCs, in their natural state, are located in the tunica media, but they migrate to the tunica intima during the early stages of atherosclerosis [70]. Once in the intima, these VSMCs begin manufacturing large amounts of ECM components, including HA [71] that form intercellular ‘‘cable-like’’ structures in the ECM [72]. Interestingly, studies have demonstrated that these cable-like structures are involved in the early proinflammatory stage of atherosclerosis [73]. 

Increased aggregation of HA and versican, in vivo, tends to increase the swelling pressure of the ECM. If it will not be counteracted by fibrillar matrix components, it would predispose the artery to stenosis [74]. Links, that stabilize the association between versican and HA, may aid in the versican retention in normal and diseased arterial tissue [74].

In human re-stenotic lesions, HA appears to be concentrated in the intima and adventitia [75]. Evidence of HA accumulation in the intima of injured and aging arterial vessels is demonstrated in both human and animal studies [76]. Older rats were shown to have an increased ratio of hyaluronidase to HA at baseline and at all time points post injury [76], likely representing a role for HA and hyaluronidase in the aging vessel. Sadowitz et al. [77] showed that, in the two processes most responsible for vessel renarrowing such intimal hyperplasia and wound contracture, HA has an integral and early role in the development and structure of re-stenotic lesions after vascular instrumentation. These observations are consistent with increased vascular stiffness induced by HA accumulation in the aorta seen by Lorentzen et al. [78]. Interestingly, in this study, increased proliferation of vascular smooth muscle cells was also associated with increased HA accumulation.

### 3.7. Eyes

HA makes up 96% of the total GAG in the vitreous humor [79]. High molecular mass HA at high concentrations possesses viscoelastic and exclusion properties [80]. Since HA sugars bind and organize water molecules, this proteoglycan-collagen interaction results in gel formation [81], directly contributing to the supramolecular organization of the vitreous and its homeostasis and biomechanics [82]. Authors found the enormous potential of HA and other binding partners, with N-terminal and C-terminal regions of proteoglycans (such as versican), to form macromolecular complexes which play a crucial role in the structural stability and functionality of the vitreous, due to their important biological properties” [83].

Narayanan and Kuppermann [84] reported the use hyaluronidases for pharmacologic vitreolysis avoiding the complications of surgery and anesthesia-related. Dissolution of the HA and collagen complex results in decreased viscosity of the extracellular matrix [85]. HA can also act much similar to an ion-exchange resin in which an electrostatic interaction forms the basis for various HA properties, including its influence upon osmotic pressure and ion transport within the vitreous [86].

### 3.8. Kidney

Over the years, HA has emerged as an important molecule in nephrological and urological studies involving ECM organization, inflammation, tissue regeneration, and viral sensing [86]. In a healthy human kidney, very little HA is expressed, but it is accumulated in the cortical interstitium during progressive kidney disease. This increase of HA concentration stimulates the recruitment of monocytes into the interstitial space, potentiating the interstitial inflammation [87]. In several kidney diseases, elevated levels of HA in renal tissue were reported in both rat models (ischemia-reperfusion injury) and human diseases (diabetic nephropathy, renal transplant rejection and kidney stone formation) [3]. Selbi et al. [88] have demonstrated that renal proximal tubular epithelial cells surround themselves in vitro with HA in an organized pericellular matrix or ’coat’. This is associated with cell migration, pericellular HA cable-like structures formation, epithelial cell trans-differentiation, ref. [89] and facilitation of fibrotic response in the context of progressive renal disease. 

It was noted that papillary mesenchymal cells stimulate HA synthesis after an obstruction, rising the interstitial HA levels [90]. HA can form gel-like matrices that are negatively charged and able to bind crystals. This is particularly relevant in obstructive uropathy [86]. Furthermore, HA is present in kidney stones in fractions that are in disproportion to HA urinary levels [91].

Han et al. [92] found, in chronic kidney disease (CKD) of animal model, an increased expression of HA. This accumulation of HA, in the renal cortex and sclerotic vessels in CKD, was in contract with the regular HA presence, only in the renal medulla under physiologic conditions. In CKD, HA accumulation was associated with an increasing α-SMA and consequently a pro-inflammatory and fibrogenic milieu [93].

## 4. Discussion

HA is a major component of the extracellular matrix surrounding migrating and proliferating cells and therefore, it is an important component of healing or regenerating tissue. One molecule of HA may contain as many as 20,000 repeating disaccharide units in a linear array. Extensive intrachain hydrogen bonding, mutual repulsion between the negatively charged carboxylate groups, and the beta linkages between the sugar residues lend HA an inherent stiffness [94]. 

**Figure 2:**
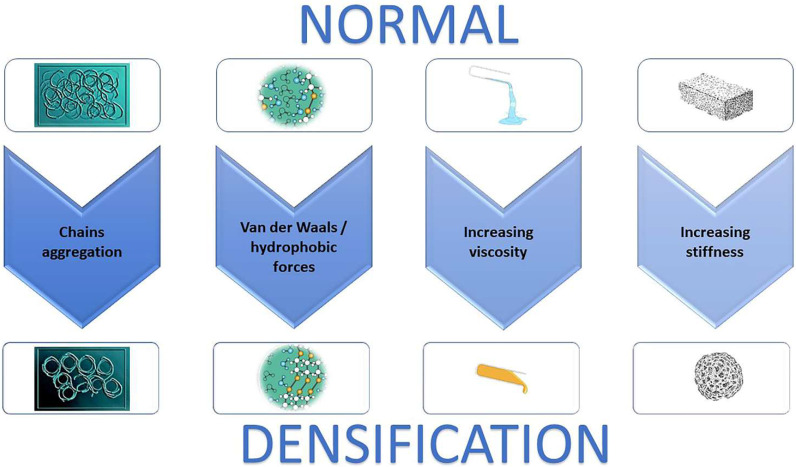
The biological process of densification development. Hyaluronan increases in concentration, then aggregates, releasing water. This process generates a macromolecular structure that increase the viscosity of the extracellular matrix consequently increasing tissue stiffness.

The large number of hydrophilic residues on the HA chain interact with water, favoring an expended coil volume, resulting in a viscous hydrated gel (Figure 2). The viscosity of HA also changes with temperature. In particular, when the temperature is increased to >40 °C, the three-dimensional superstructure of HA chains progressively breaks down [95], and consequently HA decreases in viscosity; in contrast, at low temperatures the viscosity increases. Additionally, alterations in the pH can change the viscosity of HA [96]; HA becomes more viscous in acid solution. In the muscle compartment Juel et al. [97] demonstrated that, after strenuous exercises due to the lactate accumulation, the pH can reach a value of 6.60. This could mean an increase in HA viscosity of approximately 20%, with a consequent increase in stiffness.

HA also interacts or aggregates with other large proteoglycans forming even larger complexes [50]. It was demonstrated how HA, via its association with multiple binding proteins, can be re-organized into supramolecular assemblies [98]. In the connective tissues ECM, HA binds certain proteoglycans (aggrecan, versican, and brevican) to form large complexes, that provide tissue structural integrity and mechanical function [99]. 

ECM stiffness is important for maintaining normal tissue homeostasis, and when matrix mechanics become imbalanced, disease progression may ensue [100]. 

**Figure 3:**
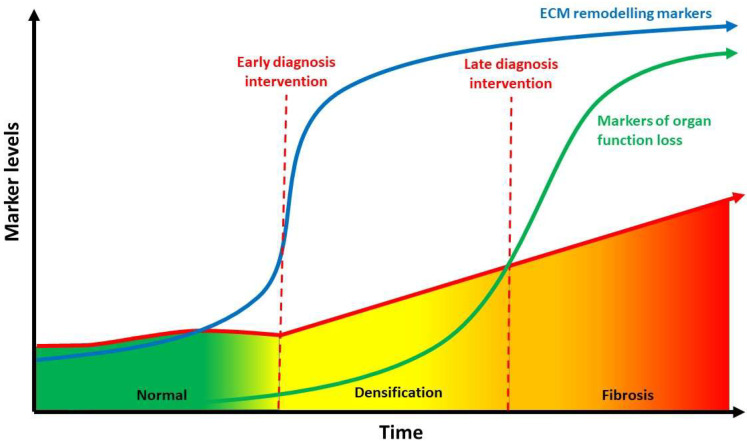
Relationship between hyaluronan concentration (indirect evaluated through ECM), densification and fibrosis over time. Biomarkers of ECM remodeling may identify molecular processes occurring in the early phases of densification, giving the opportunity for early intervention when the disease is still reversible before fibrosis sets in. ECM: extracellular matrix (Modified from [102]).

Basic imaging modality such as regular Ultrasound and MRI have not been able to provide high quality imaging of ECM stiffness. However, new modality as elasto-sonography, T1rho MRI mapping, and elasto-MRI have successfully reproduced clear images to identify this alteration [101]. These imaging may be useful to identify reversible densification versus irreversible fibrosis to help combat the clinical and economic burden of multiple diseases [102] (Figure 3). 

In addition, the fibrosis may occur as long-term consequence of densification due to an excessive collagen fibers deposition and consequent ECM remodeling. Elevated HA levels in the various fibrotic organ were reported in various studies [11,30,53,58,92], highlighting the direct association between HA accumulation and fibrosis. Moreover, the antifibrotic effect of hyaluronidase was reported in recent studies [47,77,84], whereas other studies demonstrated the role of HA synthases in the pathogenesis of fibrosis [11,30,53,58,92]. Various ECM components (hyaluronan, collagen, SPARC, α-SMA, tenascin C), that contribute to fibrosis, are overexpress in activated stromal cells (fibroblasts, myofibroblasts, stellate cells) and, in some cases, in tumor cells. Interstitial forces, that lead to blood vessel compression, are determined by a bulk of HA and collagen (predominantly types I and IV). Breakdown and synthesis of ECM components, as part of the constant remodeling of the tumor stroma, also facilitates distal metastasis. Overall, the growth of dense fibrotic tissue around tumor cells in pancreatic ductal adenocarcinomas, defined as desmoplastic reaction, impedes anti-cancer drug efficacy. This should be eliminated prior to therapy in order to avoid treatment failure [103]. 

This is a narrative review that may have excluded other organs and tissues which also demonstrated disease states associated with the accumulation of HA. Further studies should be carried out to clearly define the biological states of HA in vivo in health and disease conditions.

## 5. Conclusions

This manuscript aimed to investigate the organs that demonstrate HA aggregation in disease states, showing the importance of this phenomenon in pathological conditions. It is interesting to observe that HA accumulation and aggregation play a role in pathology of many different organs. Understanding HA aggregation will help to clarify the underlying processes and correctly differentiate between densification (HA aggregation) and fibrosis. It is critical to differentiate these in clinical practice as the therapeutic approach could be different and disease progression could be completely altered. Fortunately, differentiation is possible through imaging. The therapeutic window to treat a patient presenting densification, before fibrosis is established, is quite wide, enabling prevention to an irreversible state, which is always the best.

## Data Availability

The data presented in this study are available on request from the corresponding author.
